# Epidemiological Study of Lymphedema Prevalence and Comorbidities in Hospitalized Patients in the United States

**DOI:** 10.3390/jcm14228156

**Published:** 2025-11-17

**Authors:** Nicolas Gérard, Ioannis T. Farmakis, Luca Valerio, Lukas Hobohm, Karsten Keller, Nils Kucher, Stefano Barco, Alexandru Grigorean

**Affiliations:** 1 Department of Angiology, University Hospital Zurich, 8091 Zurich, Switzerland; nils.kucher@usz.ch (N.K.); stefano.barco@usz.ch (S.B.); 2 Center for Thrombosis and Hemostasis (CTH), University Medical Center Mainz (Johannes Gutenberg-University Mainz), 55131 Mainz, Germany; ioannis.farmakis@unimedizin-mainz.de (I.T.F.); luca.valerio@uni-mainz.de (L.V.); lukas.hobohm@unimedizin-mainz.de (L.H.); karsten.keller@unimedizin-mainz.de (K.K.); 3 Department of Cardiology, Cardiology I, University Medical Center Mainz (Johannes Gutenberg-University Mainz), 55131 Mainz, Germany; 4 Medical Clinic VII, Department of Sports Medicine, University Hospital Heidelberg, 69120 Heidelberg, Germany

**Keywords:** lymphedema, epidemiology, prevalence, NIS, Nationwide Inpatient Sample

## Abstract

**Background/Objective**: Lymphedema is a disabling condition that is both underdiagnosed and undertreated. Epidemiological data on this disease is sparse. **Methods**: The prevalence of lymphedema was studied in hospitalized patients registered in the Nationwide Inpatient Sample (NIS) of the United States (US) from 2016 to 2020. ICD-10 codes related to lymphedema were utilized to identify eligible cases. We studied comorbidity burden and outcomes during hospitalizations, including in-hospital fatality, length of stay and total charges per hospitalization. **Results**: Lymphedema was present in 0.45% (*n* = 792,475) of all hospitalizations; with prevalence increasing from 0.40% in 2016 to 0.50% in 2020. Lymphedema-mentioning hospitalizations peaked in July. The median age was 67 (IQR: 57–77) years; A total of 60% were female. Most lymphedema-mentioning hospitalizations were emergency admissions (90%). The most frequent comorbidities were arterial hypertension (77%), obesity (58%), diabetes mellitus (42%), phlegmon (38%), renal disease (32%), chronic pulmonary disease (31%), and cancer (26%). The in-hospital fatality rate was 2.3%, the median length of stay was 5 (IQR: 3–8) days, and each hospitalization incurred a median of 36,304 (IQR: 20,431, 67,171) US dollars, roughly three times higher than the average hospitalization costs in the NIS in the same period. **Conclusions**: This represents the first comprehensive nationwide study of the epidemiological and economic burden of lymphedema among hospitalized patients in the US. The findings highlight that lymphedema, although underdiagnosed, affects a significant number of patients and is associated with a considerable burden of both comorbidities and costs.

## 1. Introduction

Lymphedema is defined as a localized accumulation of protein-rich interstitial fluid that is otherwise normally drained through the lymphatic system, resulting in the swelling of the affected tissue [1,2]. Most cases of lymphedema affect the upper or lower extremities, but any body part can be involved. Primary lymphedema is caused by lymphatic dysplasia usually associated with genetic mutations, often in the context of syndromes (such as Turner or Noonan syndrome), and can be either already present at birth or manifest later in life [2,3]. It is a rare condition, with an estimated prevalence of approximately 1/100,000 in individuals aged < 20 years [4]. However, roughly 99% of all cases of lymphedema are secondary, mainly due to low-output failure from an acquired obliteration of the lymphatic drainage system by diverse causes such as infections, trauma, or malignancy, including lymphatic tumor surgery and radiotherapy [1,3]. Secondary lymphedema caused by high-output failure, when a normally functioning lymphatic system is overwhelmed by an excess of capillary fluid filtration (i.e., due to hypoalbuminemia, right heart failure, or venous insufficiency), is caused by pathologies outside the lymphatic system itself [3].

Worldwide, the most common cause of secondary lymphedema is lymphatic filariasis due to parasitic infections by Wuchereria bancrofti and Brugia malayi or timori, predominant in the tropical regions of Asia, Africa, and the Americas [5]. In Western countries, however, the most common causes of secondary lymphedema of the upper and lower extremity are considered to be iatrogenic injuries to the lymphatic system (i.e., lymph node dissection) due to the treatment of breast cancer and pelvic neoplasms, respectively [6]. Other malignancies may also cause lymphedema, either by infiltration and compression of the lymphatic system or after surgery and radiotherapy [5]. A comprehensive meta-analysis from 2010 reported an overall incidence of secondary lymphedema of 15% in cancer patients, ranging from 4 to 30% depending on the cancer type [7]. Other causes of secondary lymphedema include iatrogenic or traumatic injuries to the lymphatic system, i.e., burns or harvesting of the greater saphenous vein for aortocoronary bypass grafting—the latter with lymphedema occurring in approximately 10% of the patients [5,8]. Obesity has also been shown to cause secondary lymphedema, especially in patients with BMI > 40 kg/m^2^ [9,10].

Lymphedema can cause several complications, ranging in severity from tissue fibrosis and accumulation of adipose tissue to infections such as erysipelas and phlegmon, and is associated with reduced quality of life and functional impairment [11,12].

Lymphedema is deemed to be both underdiagnosed and undertreated [13]. Reliable data on its prevalence is currently scarce. A questionnaire-based survey of a community trust with a population of 619,000 showed a prevalence of 1.33/1000 individuals, with only 64% of those affected receiving treatment [13]. A more recent analysis reported a prevalence of 9/1000 among hospitalized patients with deep vein thrombosis in Germany [14]. Lopez et al. conducted a study including all adults admitted with a primary diagnosis of lymphedema or a primary diagnosis of extremity cellulitis and secondary diagnosis of lymphedema, registered in the NIS between 2012 and 2017, and estimated a total of 165,055 lymphedema admissions in the United States [15]. Since this work did not provide a prevalence estimate, our study addresses this gap by providing such estimates in a comparable time frame using the same database.

Eventually, more precise estimates of the prevalence of this condition are necessary to increase awareness among practitioners, to monitor secular epidemiological trends, and to support public health resource allocation, including the establishment and distribution of specialized lymphatic care centers, which may improve disease management [16].

In conclusion, the aim of this study was to assess the prevalence of lymphedema among hospitalized patients in the US, as well as the comorbidities and relevant outcomes of this population.

## 2. Methods

In this study, we analyzed data from the US Nationwide Inpatient Sample (NIS), which is part of the Healthcare Cost and Utilization Project (HCUP). The NIS is nationally representative of US community hospitals and records roughly 7 million hospitalizations per year, thus covering about 20% of all discharges from community hospitals [17]. The resulting weighted analysis thus allows to investigate about 35 million hospitalizations (100%) per year. Since 2015, diagnoses in the NIS are classified according to the International Classification of Diseases, 10th Revision (ICD-10), and procedures according to the ICD-10 Procedure Coding System (ICD-10-PCS). The NIS also provides data on in-hospital mortality, length of stay, and total charges as well as type of admission (emergency vs. elective) and type of discharge (home vs. transfer to other institution). Since all patient information in the NIS were recorded in anonymized form, approval by an ethics committee was not required to perform this analysis.

This study included all hospitalizations recorded in the NIS from January 2016 to December 2020, in which at least one of the ICD-10 codes I89.0 (lymphedema, not elsewhere classified), I97.2 (Postmastectomy lymphedema syndrome), or Q82.0 (hereditary lymphedema) was included among the discharge diagnoses. Code I89.9 (non-infective disorder of lymphatic vessels and lymph nodes, unspecified) was excluded because of its low specificity; this code may have been assigned to conditions different from lymphedema. We did not evaluate upper- and lower-extremity lymphedema, as ICD-10 codes strictly do not allow this distinction. All postmastectomy lymphedema syndromes are of course afflicting the upper extremity, yet I89.0 (lymphedema, not elsewhere classified) and Q82.0 (hereditary lymphedema) are ambiguous with respect to localization.

The general features of lymphedema-mentioning hospitalizations, including year and month, admission type (elective or emergency), and overall hospitalization costs were collected. For each hospitalization, the patient’s demographic features (age, sex, race) and concomitant diagnoses, including major comorbidities in hospitalized patients (obesity, diabetes mellitus, cardiovascular disease, cerebrovascular disease, hypertension, chronic pulmonary disease, renal disease, cancer, and dementia) were included in the analysis, as well as conditions frequently associated with lymphedema (venous thromboembolism, wounds, cutaneous/subcutaneous infections, psychiatric disorders due to substance abuse, paralysis, polyneuropathies, Turner syndrome, Noonan syndrome, and Klippel–Trenaunay syndrome). The corresponding ICD-10 codes we utilized to identify these comorbidities are listed in the supplemental tables (Supplemental Tables S1 and S2).

Outcomes included in-hospital mortality, length of stay, and patient disposition (discharge destination), as well as the most common 15 procedures performed during the hospitalization as identified by ICD-10-PCS codes. In descriptive analyses, continuous variables are presented as median and interquartile range (IQR), and categorical variables as frequency and percentages. To calculate the proportion of lymphedema cases out of all hospitalizations, the total number of hospitalizations registered in the NIS during the study period was used as the denominator. The association of demographic characteristics and comorbidities with length of stay in days was assessed using linear regression, with in-hospital death using multivariable logistic regression. The variables used in these analyses are listed in the supplemental tables (Supplemental Table S3). R (R Project for Statistical Computing, version 4.5.1) was used for the statistical analysis and figures.

## 3. Results

### 3.1. Prevalence of Lymphedema and Characteristics of Lymphedema-Mentioning Hospitalizations

**Figure 1:**
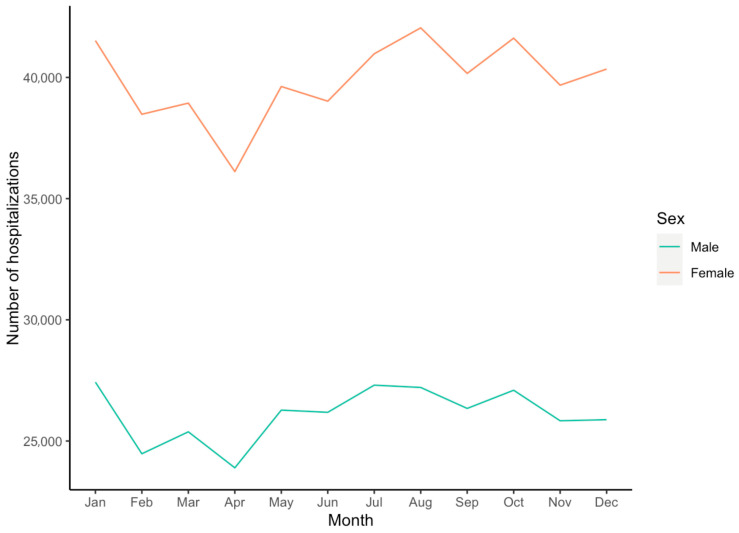
Cumulated number of lymphedema-mentioning hospitalizations for each month in the US, 2016–2020.

Out of a total number of 174,776,205 hospitalizations from 2016 to 2020, 792,475 mentioned at least one of the lymphedema ICD-10 codes as specified above, resulting in a prevalence of 4.5 per 1000 hospitalizations (0.45%) in the whole period. The prevalence of lymphedema-mentioning hospitalizations increased steadily from 0.40% in 2016 to 0.50% in 2020 (Table 1). Cumulatively over the entire study period, lymphedema-mentioning hospitalizations showed a seasonality, with a peak of hospitalizations in July and a low in April (Figure 1).

Of all lymphedema-mentioning hospitalizations, 478,735 (60%) were women. Black patients represented 15% of lymphedema-mentioning hospitalizations, whereas 74% were white, and the remaining 11% were categorized as “other”. The median age was 67 (IQR: 57–77) years, with less than 9% of patients being younger than 44 years (Table 1).

Of all lymphedema-mentioning hospitalizations, 97% were identified by ICD-10 diagnosis I89.0 (lymphedema, not elsewhere classified), while I97.2 (postmastectomy lymphedema syndrome) and Q82.0 (hereditary lymphedema) were present in 2.1% and 0.9%, respectively (Table 1). Lymphedema was the main diagnosis in 1.5% (*n* = 11,675) of all 792,475 lymphedema-mentioning hospitalizations, while the other most common main diagnosis comprised of phlegmon of the lower extremities (14.8%), sepsis (10.3%), hypertensive heart failure (7.3%), and acute renal failure (2.6%), as well as pneumonia (1.3%), chronic obstructive pulmonary disease with acute exacerbation (1.2%), and urinary tract infection (1.1%) (Supplemental Table S4).

Overall, 90% of admissions were emergencies, while the remaining 10% were elective. The median costs per lymphedema-mentioning hospitalization were 36,304 US Dollars (IQR: USD 20,431–67,171).

### 3.2. Comorbidities

The most common comorbidities (Table 1) were hypertension (77%), obesity (58%), diabetes mellitus (42%), phlegmon (38%), renal disease (32%), chronic pulmonary disease (31%), and cancer (26%). Of the latter, the most common by far was breast cancer, which was found in 2.7% of all lymphedema-mentioning hospitalizations, followed by colorectal, prostate, endometrial and ovarian cancer, each present in 0.4–0.6% of cases (Supplemental Table S1).

### 3.3. In-Hospital Outcomes

**Figure 2:**
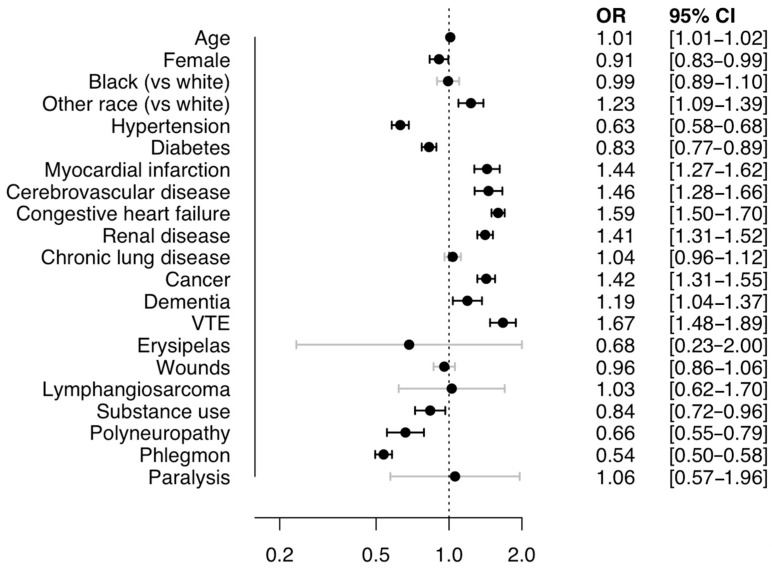
Multivariable logistic regression analysis for the association of patient demographics and associated conditions with in-hospital death among *n* = 792,475 hospitalizations with lymphedema in the US, 2016−2020: Odds ratios for in-hospital death in hospitalization with vs. without a binary covariate or for +1 numerical covariate. OR = Odds Ratio. CI = Confidence Interval. VTE = Venous thromboembolism.

In-hospital death occurred in 2.3% of lymphedema-mentioning hospitalizations and was associated, in multivariable logistic regression analysis, with concomitant venous thromboembolism (OR: 1.67, 95%CI: 1.48–1.89), cancer (OR: 1.42, 95%CI: 1.31–1.55), congestive heart failure (OR: 1.59, 95%CI: 1.50–1.70), cerebrovascular disease (OR: 1.46, 95%CI: 1.28–1.66), myocardial infarction (OR: 1.44, 95%CI: 1.27–1.62), and renal disease (OR: 1.41, 95%CI: 1.31–1.52). In contrast, a lower risk of in-hospital death was associated with concomitant phlegmon (OR: 0.54, 95%CI: 0.50–0.58), hypertension (OR: 0.63, 95%CI: 0.58–0.68), polyneuropathy (OR: 0.66, 95%CI: 0.55–0.79), substance abuse (OR: 0.84, 95%CI: 0.72–0.96), and diabetes (OR: 0.83, 95%CI: 0.77–0.89) (Figure 2).

**Figure 3:**
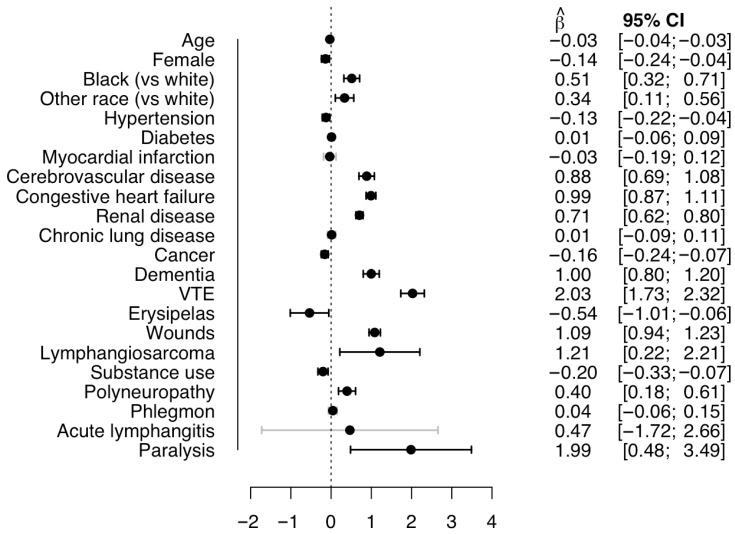
Multivariable linear regression analysis for the association between selected patient characteristics and comorbidities with days of hospital stay among *n* = 774,080 hospitalizations for lymphedema with non-fatal outcomes in the US, 2016–2020.

Linear regression analysis showed that length of stay in days increased in the presence of venous thromboembolism, heart failure, cerebrovascular disease, and renal disease, as well as non-cardiovascular conditions, i.e., dementia, wounds, lymphangiosarcoma, polyneuropathy, and paralysis (Figure 3).

Neither in-hospital death nor length of stay were associated with age or sex.

The procedures most often performed during lymphedema-mentioning hospitalizations were vascular access, tracheal intubation, gastrointestinal endoscopy, drainage of peritoneal or pleural cavity, excisions of subcutaneous tissue of the lower extremities, and excisions of toenails (Supplemental Table S5).

At discharge, 30% of cases were transferred to non–short-term hospitals, while nearly all other patients were discharged to home care (Table 2).

The beta coefficients represent the difference in days of length of stay in hospitalizations with vs. without a binary covariate of for +1 numerical covariate. VTE = Venous thromboembolism. CI = Confidence interval.

## 4. Discussion

To the best of our knowledge, this is the first study estimating the nationwide prevalence of lymphedema among hospitalizations in a contemporary Western healthcare system over five years. The use of a large nationwide dataset made it possible to analyze the occurrence and feature of a rare condition such as lymphedema, which has otherwise been difficult to study so far.

The prevalence of 4.5/1000 hospitalizations (0.45%) reflects a considerable burden in US secondary care and is comparable to that of Acute Immunodeficiency Syndrome (6/1000 hospitalizations) in the NIS in the same period [18]. This prevalence estimate aligns with recent large-scale epidemiological studies examining lymphedema burden across different healthcare systems [19]. Of note, the prevalence of lymphedema-mentioning hospitalizations out of all hospitalizations does not reflect the prevalence of lymphedema in the general population. We did not use the hospitalizations as a proxy of diagnoses to calculate a prevalence out of the general US population because a diagnosis of lymphedema does not require hospitalization. A considerable proportion of patients—presumably the majority—may be treated exclusively on an outpatient basis, either because of medical indication or for financial reasons. Consequently, using lymphedema-mentioning hospitalizations to calculate a population prevalence would have led to an underestimation of the true population prevalence possibly greater than the overestimation introduced by the possibility of repeated hospitalizations by the same patients. The calculation of a true population prevalence requires nationwide data on patients seen by general practitioners or specialist outpatient clinics and is not currently feasible until such data becomes available.

While women accounted for 56% of all hospitalizations in the NIS over the entire study period, in lymphedema-mentioning hospitalizations, 60% were females. This difference of 4% is very close to the 3% of lymphedema-mentioning hospitalizations associated with breast cancer, ovarian cancer, and endometrial cancer. Thus, sex-specific cancer might entirely explain the increased prevalence of lymphedema in women.

Lymphedema-mentioning hospitalizations showed a seasonal trend, with a peak in summer (Figure 1). This may be explained by an increase in blood flow in the skin and subcutaneous tissue due to higher temperatures in summer, thus resulting in higher lymphatic load and worsening of the edema, as studies suggest [20]. Another reason might be the reduced use of compression garments, which is often reported by patients in our clinical practice; yet, no evidence can currently be found in the literature to support this.

Compared to overall hospitalizations in the US, those with lymphedema seem to be more often accompanied by common systemic comorbidities. Lymphedema-mentioning hospitalizations showed a higher proportion of arterial hypertension (77% vs. 53%), obesity (58% vs. 17%), diabetes mellitus (42% vs. 26%), renal disease (32% vs. 16%), and chronic pulmonary disease (31% vs. 21%), compared to the prevalence of comorbidities in all inpatients registered in the NIS in 2019 as reported by Owens et al. [18]. The proportion of hospitalizations associated with cancer, considered to be the most common cause of secondary lymphedema in the US, was more than three times higher (26% vs. 7.3%).

The notably high rates of phlegmon (38%) and wounds (16%) may be interpreted in the context of subcutaneous infections, both causing secondary lymphedema and complicating pre-existing lymphedema. Moreover, subcutaneous infections and infected wounds, in the presence of underlying edema, often require intravenous antibiotic treatment and therefore hospital admission. Indeed, sepsis and phlegmon combined were the main diagnosis in (15.1%) of lymphedema-mentioning hospitalizations, explaining to some extent the high proportion of emergency admissions (90%).

Substance use was notably more prevalent in this study population (13%) than in the overall hospitalizations recorded in the NIS in the year 2019 (5.2%) [18]. Even though a direct association between intravenous drug use and lymphedema is not described in the literature, it is clinically plausible that intravenous drug use bears the risk of subcutaneous infection as well as superficial vein thrombosis. Both can cause secondary lymphedema, the former by destruction of lymphatic tissue (low-output failure), the latter by increasing lymphatic load (high-output failure).

In-hospital death is associated with acute conditions like venous thromboembolism, stroke, and myocardial infarction as well as chronic illnesses as cancer, heart failure, and renal disease. Moreover, in-hospital mortality appeared to be independent of age in our multivariate logistic regression analysis (Figure 2). This unexpected finding may be explained by collider stratification bias. Specifically, several variables associated with both age and mortality (such as cancer or advanced chronic conditions) were included as covariates in our model. Adjusting for these variables can induce a statistical artifact that masks or distorts the true relationship between age and the outcome. In this context, conditioning on intermediate variables or common effects of both age and mortality may have attenuated the observed association. We therefore caution that the absence of an age effect in our model should not be interpreted as evidence of its lack of prognostic relevance in clinical practice. Also unexpected is the pronounced inverse association of phlegmon with in-hospital death (OR: 0.54, 95%CI: 0.50–0.58). While the data does not allow for a firm conclusion, it can be speculated that hospitalizations in which phlegmon was noted as a diagnosis were more likely to have been determined by the need for intravenous treatment of phlegmon itself, compared to hospitalizations in which phlegmon was not noted and which were possibly prompted by indications with higher in-hospital fatality.

In a total of 792,475 hospitalizations, 121,240 procedures were performed, most of them not specifically related to lymphedema (vascular accesses, tracheal intubation, peritoneal or pleural fluid drainage, and gastrointestinal endoscopy). In contrast, 17% of all procedures (n = 20,475) were excisions of subcutaneous tissue or toenails, which may have been performed due to infection and are thus more likely to have been directly related to lymphedema (Supplemental Table S5).

Compared to the average cost of USD 11,700 per hospital stay in the NIS as reported by Liang et al., the median charges of lymphedema-mentioning hospitalizations were roughly three times higher (USD 36,304; IQR: USD 20,431–67,171) [21]. Considering the 32% of cases being transferred to other institutions at discharge, these findings might indicate an association of lymphedema with complex hospitalizations and a considerable burden on the healthcare system.

Recent research on lymphedema in the NIS reported lower outcomes, i.e., mortality (0.03% vs. 2.3%), length of stay (3.61 vs. 5.0 days), and costs (median USD 6180 vs. USD 36,304), while exclusively examining hospitalizations with a primary diagnosis of lymphedema or a primary diagnosis of extremity cellulitis and secondary diagnosis of lymphedema [15,22]. Yet, in this work, all lymphedema-mentioning hospitalizations were examined, including more complex cases, i.e., presenting with sepsis. Thus, including more complex cases might explain higher costs, mortality, and length of stay in our results.

Some limitations of our analysis should be noted. First, the diagnosis of lymphedema is predominantly clinical, and correctly distinguishing it from other types of edema can be challenging [16]. Sensitivity and specificity of clinical signs in predicting lymphoscintigraphy-confirmed lymphedema, which is the gold standard, are reported to be 17% and 88%, respectively [23]. Thus, a bias trending to miss many lymphedema diagnoses must be assumed. Yet, a smaller proportion of cases coded as lymphedema might have been in fact edema of some other origin, i.e., venous edema.

Second, since lymphedema was in most cases (98.5%) not the main diagnosis, i.e., leading to hospitalization, it may not have been documented as comprehensively. Hence, a potential bias of misclassification may result, with a trend to miss lymphedema. Despite these biases, in this work we decided to include all lymphedema-mentioning hospitalizations, main diagnosis or not, to allow an estimate of lymphedema prevalence in a large-scale cohort to the best of current possibilities. These limitations we deem inherent to investigating such a challenging and mostly clinically diagnosed condition as lymphedema in the context of code-based databases.

Another major limitation of this study is the inability to assess the clinical severity of lymphedema, as the NIS database does not include specific staging criteria or clinical measurements. It is likely that patients hospitalized with lymphedema, particularly when listed as a primary diagnosis, represent individuals with more advanced or symptomatic disease, which may influence the observed comorbidity profiles and healthcare utilization.

Ultimately, our findings are based on descriptive analyses of hospitalizations coded for lymphedema and do not include a matched control group of patients without lymphedema. While a comparative analysis could further contextualize the burden of disease, such an approach would require extensive additional data processing and methodological adjustments beyond the scope of this study.

## 5. Conclusions

To the best of our knowledge, this study provides the first estimate on the nationwide prevalence of lymphedema among hospitalizations, comorbidities, and other associated conditions in lymphedema-mentioning hospitalizations, and their clinical outcome. The results highlight that, even though lymphedema is an underdiagnosed and undertreated condition, it is present in a considerable number of hospitalizations and is typically associated with multiple comorbidities as well as an increased economic burden.

## Figures and Tables

**Table 1:** Baseline characteristics.

Characteristic	All Lymphedema Patients 2016–2020 *n* = 792,475
Prevalence each Year (*n/N*<; %)	
2016	141,865/35,675,421 (0.40%)
2017	150,260/35,798,453 (0.42%)
2018	161,545/35,527,481 (0.45%)
2019	176,295/35,419,023 (0.50%)
2020	162,510/32,355,827 (0.50%)
Age (years, median; IQR)	67 (57, 77)
Age (categorical)	
<18	2520 (0.3%)
18–44	65,310 (8.2%)
45–64	452,565 (57%)
≥65	272,030 (34%)
Female	478,735 (60%)
Race	
Black	121,965 (15%)
White	585,765 (74%)
Other ^1^	84,745 (11%)
Comorbidities	
Hypertension	608,005 (77%)
Obesity	456,555 (58%)
Diabetes mellitus	330,690 (42%)
Phlegmon	298,700 (38%)
Renal disease	250,025 (32%)
Chronic pulmonary disease	248,165 (31%)
Cancer	206,690 (26%)
Wounds	124,755 (16%)
Psychiatric disorders due to substance use	100,720 (13%)
Venous thromboembolism	48,110 (6.1%)
Pulmonary embolism	14,370 (1.8%)
Polyneuropathies	43,260 (5.5%)
Dementia	40,000 (5.0%)
Cerebrovascular disease	36,845 (4.6%)
Erysipelas	1600 (0.2%)
Other paralysis	2150 (0.3%)
Lymphangiosarcoma	2005 (0.3%)
Klippel–Trenaunay syndrome	550 (<0.1%)
Turner syndrome	380 (<0.1%)
Acute lymphadenitis	130 (<0.1%)
Noonan syndrome	60 (<0.1%)
Type of lymphedema	
Lymphedema, NEC (I89.0)	768,500 (97%)
Postmastectomy lymphedema syndrome (I97.2)	16,855 (2.1%)
Hereditary lymphedema (Q82.0)	7440 (0.9%)
Type of admission	
Emergency	708,425 (90%)
Elective admission	82,990 (10%)

^1^: Includes Hispanic, Asian, or Pacific Islander, Native American, and “other”.

**Table 2:** Outcomes.

Characteristic	All Patients, *n* = 792,475
In-hospital mortality	18,065 (2.3%)
Patient disposition	
Discharged to home or self-care	313,230 (40%)
Transfer: other type of facility ^1^	236,290 (30%)
Home care	201,570 (25%)
Transfer: short-term hospital	16,075 (2.0%)
Against medical advice	6780 (0.9%)
Discharged alive, destination unknown	90 (<0.1%)
Length of stay (days, median; IQR)	5.0 (3.0, 8.0)
Total charges (USD, median; IQR)	36,304 (20,431, 67,171)

^1^: All “none-acute care hospital” healthcare facilities, i.e., nursing homes, rehabilitation facilities.

## Data Availability

The original contributions presented in the study are included in the article (and Supplementary Material), further inquiries can be directed to the corresponding author.

## Supplementary Materials

The following supporting information can be downloaded at: https://www.mdpi.com/article/10.3390/jcm14228156/s1, Supplemental Table S1: Cancer diagnoses; Supplemental Table S2: ICD-10 codes corresponding to comorbidities in Table 1 and in Figure 2 and Figure 3; Supplemental Table S3: Variables used for multivariate analyses in Figure 2 and Figure 3; Supplemental Table S4: 10 most common main diagnosis in all lymphedema-mentioning hospitalizations; Supplemental Table S5: Fifteen most common interventions.

## Abbreviations

The following abbreviations are used in this manuscript:

BMI	Body mass index
CI	Confidence interval
ICD-10	International Classification of Disease, 10th Revision
ICD-10-PCS	ICD-10 Procedure Coding System
IQR	Interquartile range
NEC	Not elsewhere classified
NIS	Nation Wide Inpatient Sample
OR	Odds ratio
US	United States
USD	United States Dollar
VTE	Venous thromboembolism
