# Supplementary material for: Epidemiological Study of Lymphedema Prevalence and Comorbidities in Hospitalized Patients in the United States
Source: J Clin Med. 2025 Nov 17;14(22):8156. doi: 10.3390/jcm14228156 (PMC12653852; doi:10.3390/jcm14228156)
Supplement: Supplementary file 1 [file jcm-14-08156-s001.zip › jcm-3863089-supplementary.pdf]

**Supplemental Table S1: Cancer diagnoses**

| **Characteristic (ICD-10 code)** | **All patients, N = 792,475** |
|---|---|
| Breast cancer (C50.*) | 21,450 (2.7%) |
| Colorectal cancer (C18.*, C19.*, C20.*, C21.*) | 4,535 (0.6%) |
| Prostate cancer (C61.*) | 4,380 (0.6%) |
| Endometrial cancer (C54.*, C55.*) | 4,190 (0.5%) |
| Ovarian cancer (C56.*) | 2,870 (0.4%) |
| Bladder cancer (C67.*) | 2,080 (0.3%) |
| Melanoma (C43.*) | 2,015 (0.3%) |
| Cervical cancer (C53.*) | 1,440 (0.2%) |
| Head and neck cancer (C0.*, C1[01234].*) | 1,435 (0.2%) |
| Vulval cancer (C51.*) | 695 (<0.1%) |

**Supplemental Table S2: ICD-10 codes corresponding to comorbidities in Table 1 and in Figures 2 and 3**

| Condition | utilized ICD-10 codes |
|---|---|
| Hypertension | I10.*, I11.*, I12.*, I13.* |
| Obesity | E66.[01289] |
| Diabetes mellitus | E10.*,E11.*, E13.* |
| Chronic pulmonary disease | I27.8, I27.9, J6[01234567].*, J68.4, J70.1, J70.3 |
| Myocardial infarction | I21.*, I22.*I25.2 |
| Congestive heart failure | I09.9, I11.0, I13.0, I13.2 I25.5, I42.[056789], I43.*, I50.* |
| Renal disease | I12.0, I13.1, N03.*, N05.*, N18.*, N19.* |
| Cancer | C*.*, D0*.*, D3[7-9].*, D4[0-5].*, Z85.* |
| Cerebrovascular disease | G45.*, G46.*, H34.0, I6*.* |
| Dementia | F0[0123].*, F051.*, G30.*, G31.1 |
| VTE | I26.*, I80.*, I82.* |
| PE | I26.* |
| Phlegmon | L03.* |
| Wounds | L97.*, L98.[489] |
| Erysipelas | A46.* |
| Acute lymphadenitis | L04.* |
| Lymphangiosarcoma | C49.* |
| Psychiatric disorders due to substance use | F1[1-9].* |
| Polyneuropathies | G6[0-4].* |
| Other paralysis | G83.* |
| Turner syndrome | Q96.* |
| Noonan syndrome | Q87.1 |
| Klippel-Trenaunay syndrome | Q87.2 |

**Supplemental Table S3: Variables used for multivariate Analyses in Figure 2 and 3**

| **Comorbidities** | As listed in Table S2 with corresponding ICD-10 codes |
|---|---|
| **Cancerdiagnoeses** | As listed in Table S1 with corresponding ICD-10 codes |
| **HCUP Data Element** | **Descriptive Title** |
| AGE | Age in years at admission |
| AGE_NEONATE | Neonatal age (first 28 days after birth) indicator |
| AMONTH | Admission month |
| AWEEKEND | Admission day is on a weekend |
| DIED | Died during hospitalization |
| DISPUNIFORM | Disposition of patient, uniform coding |
| DQTR | Discharge quarter |
| DRG | DRG in use on discharge date |
| DRG_NoPOA | DRG in use on discharge date, calculated without POA |
| DRGVER | DRG or MS-DRG grouper version used on discharge date |
| DXVER | Diagnosis codes ICD version indicator |
| ELECTIVE | Elective versus non-elective admission |
| FEMALE | Indicator of sex |
| H_CONTRL | Control/ownership of hospital |
| HCUP_ED | HCUP indicator of emergency department record |
| HOSP_BEDSIZE | Bedsize of hospital |
| HOSP_DIVISION | Census Division of hospital (STRATA) |
| HOSP_LOCTEACH | Location/teaching status of hospital |
| HOSP_NIS | NIS hospital number |
| I10_DXn1-40 | ICD-10-CM Diagnosis |
| I10_NDX | Number of ICD-10-CM diagnoses on this discharge |
| I10_NPR | Number of ICD-10-PCS procedures on this discharge |
| I10_PR1-25 | ICD-10-PCS Procedure |
| KEY_NIS | NIS record number |
| LOS | Length of stay, cleaned |
| MDC | MDC in effect on discharge date |
| MDC_NoPOA | MDC in use on discharge date, calculated without POA |
| NIS_STRATUM | Stratum used to post-stratify hospital |
| PAY1 | Expected primary payer, uniform |
| PL_NCHS | Patient Location: NCHS Urban-Rural Code |
| PRDAYn | Number of days from admission to procedure n |
| PRVER | Procedure codes ICD version indicator |
| RACE | Race/ethnicity of patient |
| TOTCHG | Total charges, cleaned |
| TRAN_IN | Indicator of a transfer into the hospital |
| TRAN_OUT | Transfer out indicator |
| YEAR | Calendar year |
| ZIPINC_QRTL | Median household income for patient's ZIP Code (based on current year) |

**Supplemental Table S4: 10 most common main diagnosis in all lymphedema-mentioning hospitalizations**

| # | All patients, N = 792,475 | n (%) |
|---|---|---|
| 1 | Sepsis, unspecified (A41.9) | 81735 (10.31%) |
| 2 | Cellulitis of left lower limb (L03.116) (Syn.= Phlegmon) | 62400 (7.87%) |
| 3 | Cellulitis of right lower limb (L03.115) (Syn.= Phlegmon) | 55150 (6.96%) |
| 4 | Hypertensive heart and renal disease with (congestive) heart failure (I13.0) | 33075 (4.17%) |
| 5 | Hypertensive heart disease with (congestive) heart failure (I11.0) | 25030 (3.16%) |
| 6 | Acute renal failure, unspecified (N17.9) | 20845 (2.63%) |
| 7 | Lymphoedema, not elsewhere classified (I89.0) | 11675 (1.47%) |
| 8 | Pneumonia, unspecified (J18.9) | 10650 (1.34%) |
| 9 | Chronic obstructive pulmonary disease with acute exacerbation, unspecified (J44.1) | 9795 (1.24%) |
| 10 | Urinary tract infection, site not specified (N39.0) | 8975 (1.13%) |

**Supplemental Table S5: 15 most common interventions**

| # | All patients, N = 792,475 | n (%) |
|---|---|---|
| 1 | Insertion of Infusion Device into Superior Vena Cava, Percutaneous Approach | 42570 (5.4 %) |
| 2 | Insertion of Endotracheal Airway into Trachea | 42570 (1.9%) |
| 3 | Excision of Toe Nail, External Approach | 10250 (1.3%) |
| 4 | Inspection of Upper Intestinal Tract | 6335 (0.8%) |
| 5 | Insertion of Infusion Device into Right Atrium, Percutaneous Approach | 5860 (0.7%) |
| 6 | Excision of Stomach, Via Natural or Artificial Opening Endoscopic, Diagnostic | 5685 (0.7%) |
| 7 | Insertion of Infusion Device into Right Internal Jugular Vein, Percutaneous Approach | 4605 (0.6%) |
| 8 | Insertion of Tunneled Vascular Access Device into Chest Subcutaneous Tissue and Fascia, Percutaneous Approach | 4590 (0.6%) |
| 9 | Insertion of Monitoring Device into Upper Artery, Percutaneous Approach | 4570 (0.6%) |
| 10 | Inspection of Lower Intestinal Tract | 4065 (0.5%) |
| 11 | Drainage of Right Pleural Cavity, Percutaneous Approach | 3940 (0.5%) |
| 12 | Drainage of Peritoneal Cavity, Percutaneous Approach | 3725 (0.5%) |
| 13 | Excision of Left Lower Leg Subcutaneous Tissue and Fascia, Open Approach | 3550 (0.5%) |
| 14 | Excision of Right Lower Leg Subcutaneous Tissue and Fascia, Open Approach | 3420 (0.4%) |
| 15 | Excision of Left Foot Subcutaneous Tissue and Fascia, Open Approach | 3255 (0.4%) |
